# Isolated Splenic Metastasis and Splenic Bed Recurrence From Endometrial Carcinoma: A Rare Case

**DOI:** 10.7759/cureus.83566

**Published:** 2025-05-06

**Authors:** Aasef M Mansoor

**Affiliations:** 1 Radiology, Salmaniya Medical Complex, Manama, BHR

**Keywords:** endometrial cancer, isolated metastasis, long-term surveillance, papillary serous carcinoma, postmenopausal bleeding, rare metastasis, recurrence, serous endometrial cancer, splenectomy, splenic metastasis

## Abstract

Papillary serous carcinoma is a rare and aggressive subtype of endometrial cancer with a strong predilection for extrauterine spread. Although metastasis typically involves the peritoneum, lymph nodes, and lungs, isolated splenic metastases are exceedingly rare. We report the case of a 66-year-old woman initially diagnosed with papillary serous endometrial carcinoma following an episode of postmenopausal bleeding. She underwent surgical staging with hysterectomy, bilateral salpingo-oophorectomy, and pelvic lymphadenectomy, followed by adjuvant chemotherapy. Two years later, she presented with non-specific abdominal discomfort, and imaging revealed a solitary splenic lesion. A splenectomy was performed, and histopathology confirmed metastatic papillary serous carcinoma. The patient remained stable until a further year later when she was re-presented with fatigue and left upper quadrant pain. Imaging demonstrated a soft tissue mass in the splenic bed, and histopathological analysis confirmed the recurrence of the known malignancy. This case underscores the potential for late, isolated splenic metastasis and recurrence in the splenic bed after splenectomy which is exceptionally uncommon. It highlights the importance of long-term surveillance and the consideration of atypical metastatic patterns in patients with serous endometrial cancer. This report adds to the limited literature and reinforces the value of individualized, multidisciplinary management in such complex clinical scenarios.

## Introduction

Endometrial cancer is the most common gynecologic malignancy in developed countries, with papillary serous carcinoma representing a high-grade histologic subtype that accounts for less than 10% of cases but contributes disproportionately to cancer-related mortality [1]. Papillary serous endometrial carcinoma is characterized by aggressive clinical behavior, a high propensity for extrauterine spread, and a tendency to recur despite early-stage diagnosis and comprehensive surgical staging [2]. The most frequent sites of distant metastasis include the lungs, liver, peritoneum, and lymph nodes. In contrast, the spleen is an exceedingly rare site of metastatic spread in any solid malignancy, including endometrial cancer [3].

Metastatic involvement of the spleen typically occurs as part of widespread disease; isolated splenic metastases, especially from gynecologic cancers, are extremely uncommon [3]. Among the limited cases reported in the literature, endometrial carcinoma has rarely been implicated as the primary tumor, and even fewer have involved the serous subtype. Several hypotheses have been proposed to explain the rarity of splenic metastases, including the spleen’s unique anatomical, hemodynamic, and immunological characteristics that may hinder tumor cell implantation and growth [4].

We report a rare and diagnostically challenging case of a woman with a history of papillary serous endometrial carcinoma who developed an isolated splenic metastasis nearly two years after initial treatment. This was followed by a subsequent recurrence in the splenic bed. To our knowledge, this is among the few documented cases of isolated splenic metastasis from this histologic subtype and highlights the importance of extended surveillance, awareness of unusual recurrence patterns, and individualized multidisciplinary management.

## Case presentation

A 66-year-old multiparous woman with a history of hypertension, type 2 diabetes mellitus, hyperlipidemia, and gout presented with postmenopausal bleeding of several weeks’ duration. She had undergone natural vaginal delivery and had been menopausal for two decades. She reported no associated pain or weight changes. There was no relevant family history of malignancy. On examination, her vital signs were within normal limits. Abdominal examination revealed obesity and an easily reducible incisional hernia. Speculum and bimanual pelvic examinations were unremarkable, and there were no visible cervical lesions.

**Figure 1:**
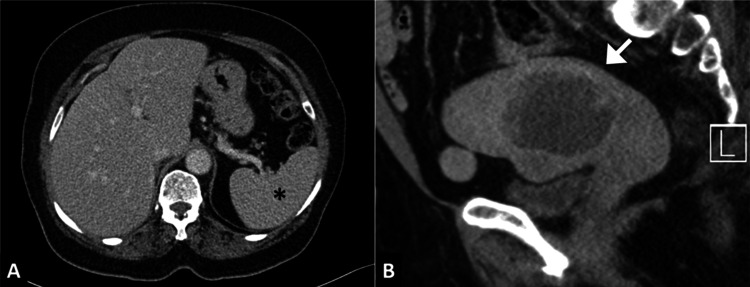
Cross-sectional imaging at initial diagnosis A. Axial contrast-enhanced CT image of the upper abdomen showing a normal-appearing spleen (asterisk). B. Sagittal pelvic CT image demonstrating a heterogeneously enhancing endometrial mass (arrow) arising from the uterine cavity, consistent with primary endometrial carcinoma.

**Figure 2:**
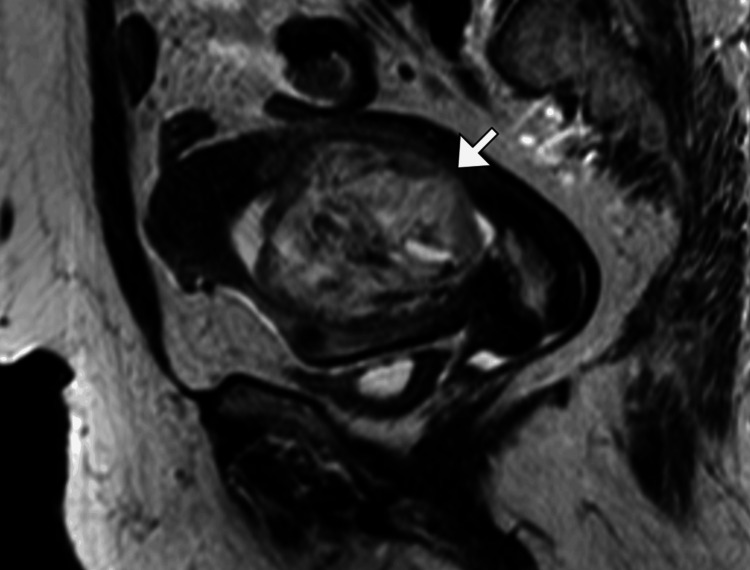
Sagittal T2-weighted MRI of the pelvis Sagittal MRI demonstrates a large endometrial mass (arrow) distending the uterine cavity with heterogeneous signal intensity and preserved endometrial–myometrial junction, consistent with papillary serous endometrial carcinoma.

Transvaginal ultrasound showed a thickened endometrium with suspicious echogenicity, and subsequent endometrial sampling revealed papillary serous endometrial carcinoma. A contrast-enhanced CT of the pelvis demonstrated a markedly distended endometrial cavity with a well-defined, heterogeneously enhancing mass measuring 8.5 × 7.6 × 5.6 cm (Figure 1). The myometrial-endometrial junction appeared preserved (Figure 2). Multiple uterine fibroids were also noted. The ovaries were not well visualized. There was no evidence of pelvic lymphadenopathy or ascites.

Following a preoperative workup, the patient underwent a total abdominal hysterectomy with bilateral salpingo-oophorectomy and pelvic lymph node dissection. Intraoperative findings included a bulky bicornuate uterus, atrophic adnexa, and bilateral pelvic lymphadenopathy. A pelvic kidney was noted, consistent with prior imaging. Histopathology confirmed grade 3 papillary serous carcinoma with lymphovascular invasion. No adjuvant therapy was administered immediately postoperatively, and she was discharged in stable condition with regular follow-up planned.

**Figure 3:**
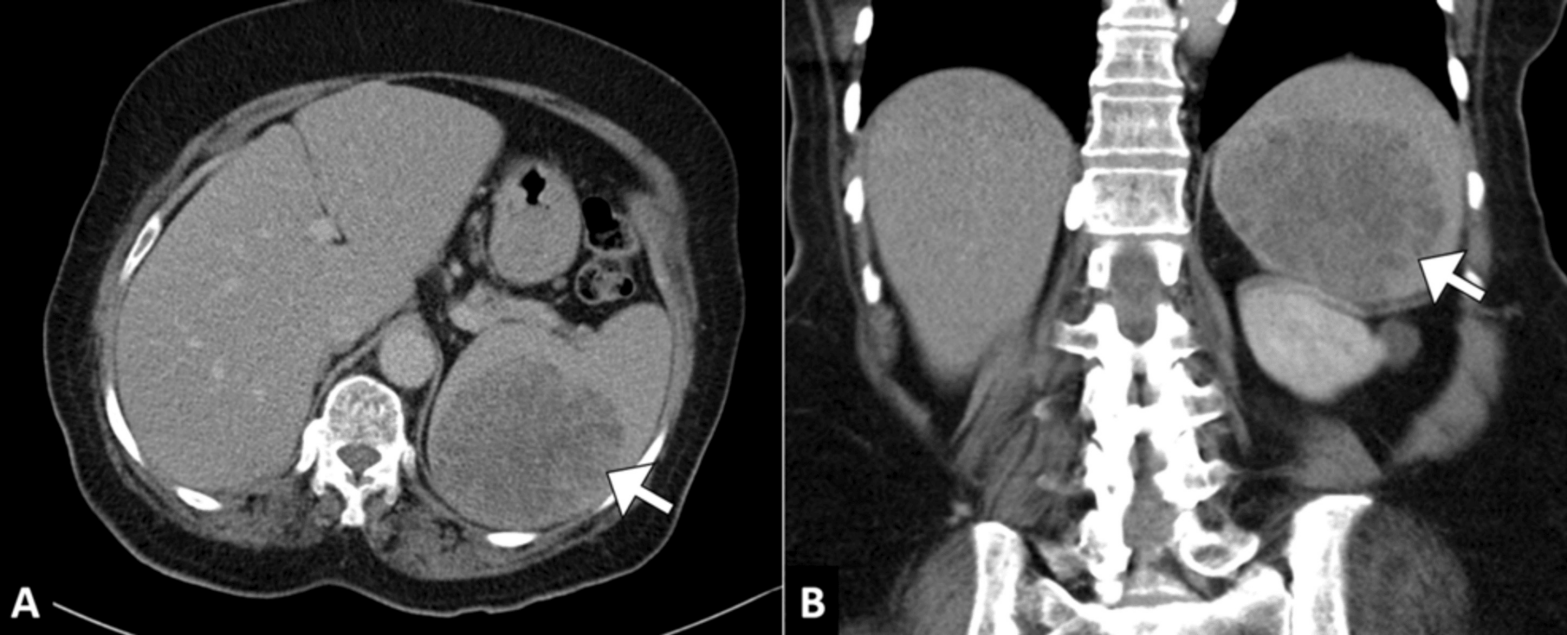
Isolated splenic metastasis on follow-up CT Axial (A) and coronal (B) contrast-enhanced CT images show a solitary, heterogeneous splenic lesion (arrow) with irregular margins and central necrosis, consistent with metastatic disease.

Approximately two years after her initial diagnosis, the patient was found to have a suspicious lesion in the spleen during routine surveillance imaging. A contrast-enhanced CT scan of the abdomen demonstrated an isolated, heterogeneous splenic mass with features concerning metastasis (Figure 3). No other visceral or nodal metastases were identified. After a multidisciplinary discussion, she underwent elective splenectomy. Postoperative recovery was uneventful.

Several months later, the patient presented to the emergency department with sepsis and hypotension requiring intensive care admission. She had developed acute kidney injury with oliguria, and hypoxia, and was intubated. On examination, she was sedated and unresponsive, with non-reactive pupils and no purposeful movements. She had no urine output for two days despite diuretic therapy. Laboratory evaluation revealed rising creatinine and urea levels, with normal lactate.

**Figure 4:**
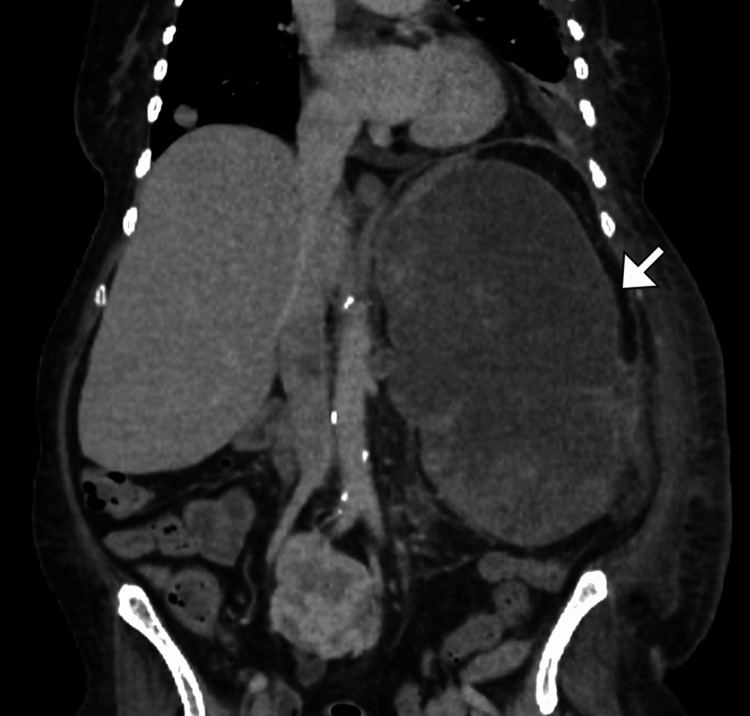
Coronal contrast-enhanced CT showing local recurrence Coronal CT image demonstrates a soft-tissue mass (arrow) in the left upper abdomen at the prior splenectomy site, consistent with recurrent disease in the splenic bed following resection of isolated splenic metastasis.

Abdominal imaging during this admission revealed recurrent soft tissue mass in the splenic bed, consistent with local recurrence (Figure 4). A retroperitoneal drain was placed, which yielded 50 mL of serosanguinous fluid. The patient was managed with broad-spectrum antibiotics, vasopressors, and supportive measures. Despite maximal supportive care, her neurologic status did not improve, and goals of care discussions were initiated with the family. Throughout her illness, she was followed closely by gynecologic oncology, infectious disease, and nephrology teams.

## Discussion

The patient’s clinical course was remarkable for a disease-free interval of approximately two years following primary surgical management of papillary serous endometrial carcinoma, after which she developed an isolated splenic metastasis. Imaging detected a solitary splenic lesion in the left upper quadrant, and splenectomy confirmed metastatic high-grade serous carcinoma. This solitary metastasis was the first and only site of recurrence at that time. However, several months post-splenectomy she experienced a local recurrence at the splenic fossa, confirmed by histopathology. Such a delayed isolated recurrence is consonant with prior reports; literature reviews note that splenic metastases from endometrial cancer typically manifest after a long latent period (ranging from under a year to >10 years) and are often the first indication of tumor relapse [5,6].

Metastatic involvement of the spleen by solid tumors is uncommon (autopsy series suggest an overall incidence of only ~2-7%) [3]. Within this context, endometrial carcinoma metastasizing to the spleen is exceptionally rare [5]. A case review in a major registry remarked that only 13 such cases had been described through 2019 [6]. Notably, almost all reported splenic metastases arose from endometrioid endometrial cancers; only a single previously published patient with high-grade serous (papillary serous) histology has been identified [3].

Several hypotheses have been advanced to explain why the spleen is a so-called “hostile soil” for metastatic seeding [7]. Reviews emphasize that anatomic and microenvironmental factors likely account for the rarity of splenic metastases. Anatomically, the acute angle of the splenic artery of the celiac axis may mechanically impede embolic tumor cells from entering the spleen. The spleen’s unique circulation - with sinusoidal vasculature that rhythmically contracts - is thought to facilitate the clearance of circulating tumor cells [7]. Furthermore, the spleen has no afferent lymphatic channels, so lymphogenous spread is extremely unlikely; most splenic metastases (when they occur) are thought to arise via hematogenous routes [7].

Published case reports of endometrial cancer with solitary splenic recurrence commonly involved middle-aged women (mean age ~57 years) with prior hysterectomy and adnexectomy [3]. Most reported patients initially had the early-stage disease (10 of 17 cases were stage I) treated with a total hysterectomy and bilateral salpingo-oophorectomy. In these series, the majority of splenic metastases arose metachronously (often several years later) rather than synchronously and were histologically limited to the splenic parenchyma [3,6]. For example, one review noted all previously reported cases of isolated splenic metastasis were metachronous, solitary, and confined to the splenic tissue. Symptomatology in the literature was variable: some patients had vague left upper-quadrant pain or splenomegaly, whereas others were asymptomatic and detected only by surveillance imaging. Follow-up outcomes were generally favorable when the splenic lesion was completely resected; many patients reported “no evidence of disease” at the last follow-up after splenectomy (often combined with adjuvant therapy) [3,6].

Recurrence of disease in the splenic bed after splenectomy - as occurred in this patient - is exceptionally unusual and poses management challenges. Possible mechanisms include microscopic residual tumor left at the splenectomy site or rapid re-seeding. In the absence of other metastases, options include reoperation (though anatomy is difficult post-splenectomy), localized radiotherapy, or systemic therapy escalation. In analogous situations (e.g., colorectal splenic metastasis), some surgeons have recommended re-exploration or targeted radiation to the splenic remnant.

## Conclusions

This case highlights several lessons for practice. First, clinicians should recognize that endometrial carcinoma can metastasize to very uncommon sites even after a prolonged disease-free interval. Because splenic metastases are often asymptomatic, routine surveillance imaging should include the upper abdomen - especially in high-risk histologies like serous carcinoma. Standard follow-up protocols often focus on the pelvis and chest, but the inclusion of abdominal imaging may permit earlier detection of unusual recurrences. Second, the extraordinarily late and isolated nature of splenic relapse in endometrial cancer warrants extended follow-up beyond the typical five-year window.
